# Respiratory Parameters as Predictors of Balance and Gait Ability in Patients with Stroke at Discharge

**DOI:** 10.3390/ijerph20237098

**Published:** 2023-11-22

**Authors:** Hee-Yong Park, Oh-Yun Kwon, Chung-Hwi Yi, Hye-Seon Jeon, Woochol Joseph Choi, So-Young Ahn, Ui-Jae Hwang

**Affiliations:** 1 Department of Rehabilitation Medicine, Chungnam National University Hospital, Daejeon 35015, Republic of Korea; hee_yong_@cnuh.co.kr (H.-Y.P.); asyoung@cnuh.co.kr (S.-Y.A.); 2 Department of Physical Therapy, The Graduate School, Yonsei University, Wonju 26493, Republic of Korea; 3 Department of Physical Therapy, College of Software and Digital Healthcare Convergence, Yonsei University, Wonju 26493, Republic of Korea; pteagle@yonsei.ac.kr (C.-H.Y.); hyeseonj@yonsei.ac.kr (H.-S.J.); wcjchoi@yonsei.ac.kr (W.J.C.); hwangu33@nate.com (U.-J.H.); 4 Kinetic Ergocise Based on Movement Analysis Laboratory, Yonsei University, Wonju 26493, Republic of Korea

**Keywords:** balance, gait, pulmonary function test, respiratory muscle strength, stroke

## Abstract

Pulmonary complications are frequent in stroke, contributing to both mortality and morbidity rates. Respiratory parameters in such patients encompass both pulmonary function and respiratory muscle strength. Identifying respiratory function variables that influence the balance and gait ability of patients with stroke is crucial for enhancing their recovery in these aspects. However, no study has assessed predictions for a comprehensive array of balance and gait abilities in such patients. We aimed to examine whether initial respiratory muscle strength and pulmonary function can predict balance and gait ability at discharge from a rehabilitation program. Thirty-one patients with stroke were included in this prospective observational study. Multiple regression models with a forward selection procedure were employed to identify respiratory parameters (including peak expiratory flow and maximal expiratory pressure) that contributed to the results of balance assessments and gait evaluations at the time of discharge. The peak expiratory flow (PEF) served as a predictor explaining 42.0% of the variance. Similarly, the maximal expiratory pressure (MEP) was a predictor variable explaining 32.0% of the variance. PEF and MEP assessments at the initial stage as predictive factors for both balance and gait ability are important in stroke management.

## 1. Introduction

Stroke is recently the second most common cause of death and a leading cause of severe long-term disability worldwide [1]. Stroke is a type of brain damage that affects trunk and limb muscle function. Weakness in trunk muscles and diminished trunk position sense among patients with stroke hinder trunk control, negatively impacting postural stability. Furthermore, such impairments extend to respiratory muscles [2,3,4]. Post-stroke, the respiratory muscles, which are crucial for trunk control, tend to weaken in tandem with trunk muscles. This weakening, combined with diminished respiratory function and exercise capacity, leads to difficulty in performing daily activities [5]. 

Pulmonary complications are frequent in the early stages following ischemic stroke, significantly contributing to both mortality and morbidity rates [6]. Patients with severe neurological diseases frequently experience respiratory complications, such as ventilator-associated pneumonia, acute respiratory distress syndrome, and neurogenic pulmonary edema. Respiratory failure, co-occurring with severe neurological disorders, can increase mortality risk and worsen neurological prognosis [7]. Ventilator-associated pneumonia occurs in 20–50% of subarachnoid hemorrhage cases [8], and the incidence of acute respiratory distress syndrome is 14–27% for subarachnoid hemorrhage, 27% for intracerebral hemorrhage, and 4% for ischemic stroke [9,10]. Neurogenic pulmonary edema affects 2–29% of patients with subarachnoid hemorrhage [11]. Additionally, patients with stroke could develop aspiration pneumonia due to a combined impaired swallowing and a weakened cough reflex, which carries the risk of leading to a poor outcome [12]. Initial post-stroke phases might not immediately reflect respiratory function abnormality. However, progressive decreases in vital capacity (VC), forced vital capacity (FVC), forced expiratory volume in 1 s (FEV_1_), and peak expiratory flow (PEF) have been reported [13]. Furthermore, Teixeira-Salmela et al. [14] demonstrated that the maximal expiratory pressure (MEP) and maximal inspiratory pressure (MIP) are significantly lower in chronic stroke survivors than in age-matched healthy individuals, indicating a link between respiratory muscle weakness and lung function impairment.

Post-stroke, the central control of respiration can be compromised, resulting in various respiratory disorders. Reduced chest movement, respiratory muscle weakness, disrupted muscular synergy, and abnormal trunk postural control contribute to this phenomenon [15]. Previous studies have reported that activating impaired respiratory muscles may compromise core stability, which is essential for mobility and balance [16,17]. Although previous studies have primarily focused on the correlation between respiratory function and balance assessment [2,18], none have assessed predictions for gait abilities in patients with stroke.

The respiratory parameters in patients with stroke encompass both pulmonary function (FVC and PEF) and respiratory muscle strength (MIP and MEP). Examining distinct respiratory function variables that impact the balance and gait ability of patients with stroke is vital for enhancing their recovery in these aspects. This can be a useful therapeutic strategy for the rehabilitation of patients with stroke. Therefore, in this study, we aimed to determine whether initial respiratory muscle strength and pulmonary function can predict balance and gait ability upon discharge.

## 2. Materials and Methods

### 2.1. Study Design and Samples

This prospective observational study included patients from Chungnam National University Hospital in South Korea from June 2022 to August 2023. A priori power analysis was performed using G* Power software (ver. 3.1.2; Franz Faul, University of Kiel, Kiel, Germany) based on the findings of a pilot study predicting TIS involving 30 patients with stroke. The sample size was calculated with a power of 0.80, an alpha level of 0.05, and an effect size of 0.569, which was determined by computing the correlation coefficient (R^2^ = 0.363) with four predictors. The analysis indicated that a minimum of 27 participants would be required for multiple regression analysis. 

**Figure 1:**
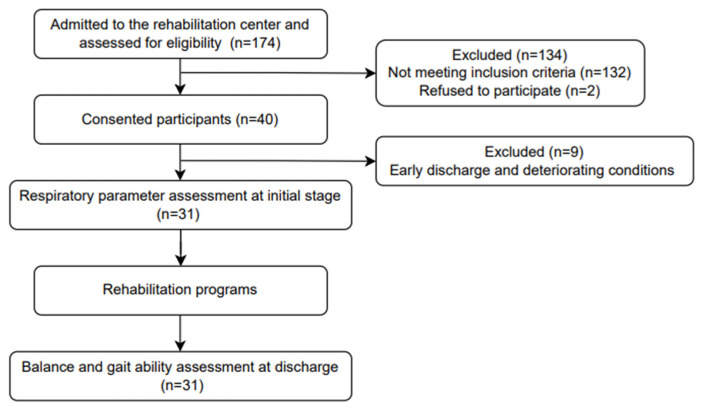
Flow chart for participants’ recruitment and experimental procedures in this study.

The participants were recruited from Chungnam National University Hospital, and prior to testing, the research procedure was thoroughly explained to the participants and voluntary informed consent was obtained. Figure 1 shows the flow chart for participants’ recruitment and experimental procedures in this study. This study was approved by the Institutional Review Board of Chungnam National University (CNUH 2022-04-005) and was registered at the Clinical Research Information Service (CRIS) in the Republic of Korea (trial unique registration number: KCT0007396).

### 2.2. Inclusion and Exclusion Criteria

The inclusion criteria were as follows: (1) patients with a clinical diagnosis of hemorrhage or ischemic stroke; (2) inpatients within 6 months of stroke onset; (3) patients aged between 60 and 80 years; (4) patients willing to voluntarily participate in the pulmonary function test (PFT); (5) patients with the cognitive ability to perform PFT as indicated by scores of more than 12 on the Korean version of the mini-mental state examination (K-MMSE); and (6) patients with the functional ability to sit on the back in a wheelchair. The exclusion criteria encompassed the following: (1) individuals with medical conditions that could potentially influence PFT outcomes, such as chronic obstructive and restrictive airway disease, bronchial asthma, aspiration pneumonia, congestive cardiac disease, or oropharyngeal cancer; (2) individuals with serious neurological disorders other than stroke; (3) individuals taking medications that may induce drowsiness or interfere with neuromuscular control; (4) those with recent eye surgery, open heart surgery, abdominal surgery, myocardial infarction, pneumothorax, pulmonary embolism, or aortic aneurysm; (5) individuals with severe hypertension (systolic blood pressure > 200 mmHg, diastolic blood pressure > 140 mmHg); and (6) those with tracheostomy status [18,19].

### 2.3. Procedures

#### 2.3.1. Respiratory Variable Assessments

Pulmonary function and respiratory muscle strength assessments were conducted following the guidelines established by the European Respiratory Society/American Thoracic Society in 2002 [20]. The respiratory variables were measured at the commencement of the conventional stroke rehabilitation program.

To evaluate pulmonary function, a spirometer (MasterScreen Pneumo Jaeger, Wurzburg, Germany) was employed. The FVC and PEF values were recorded in liters and L/s. To measure the FVC and PEF, the patients were instructed to take maximal inspiration followed by a rapid exhalation. The forced exhalation was sustained for at least 6 s, with a complete and rapid inhalation at the end of the exhalation phase. The patient performed the procedure more than three times, and the examiner selected the highest value [18,21].

The measurement of inspiratory and expiratory muscle strength involved assessing the pressure generated at the mouth level during maximal respiratory efforts. A microspirometer (Micro RPM, Micro Medical Ltd., Kent, UK) was utilized to measure respiratory muscle strength (MIP and MEP). The patients were instructed to inhale or exhale through the mouthpiece while maintaining maximal pressure. The data were recorded when the maximum pressure was maintained for at least 1 s, ensuring a difference of no more than 5% between the two maximum values. The highest value among the three measurements was retained. This highest value was then presented in centimeters of water (cmH_2_O) and expressed as a percentage of the normal value based on sex and age [21].

#### 2.3.2. Balance and Gait Variable Assessments

At the point of discharge from the rehabilitation program, balance and gait were assessed. The participants were advised to wear comfortable, suitable clothing and shoes and prepare for the potential use of walking aids (canes and walkers). The testing area was equipped with treatment tables and chairs, and when a fall risk was present, the examiner provided the necessary supervision and assistance.

Trunk balance ability was evaluated using the trunk impairment scale (TIS), which has a score range of 0 to 23. A higher score indicates improved control of trunk movement, encompassing assessments of static and dynamic sitting balance as well as coordination [22].

For a quantitative assessment of balance and fall risk determination, the Berg balance scale (BBS) was employed. For assessment using the BBS, sufficient space is required, and the necessary equipment includes a stopwatch, chair, ruler, and step. The BBS consists of 14 items, such as sitting, standing, and changing positions, and in most items, the position must be maintained for a specified period of time. It measures both the static and dynamic abilities of balance. The BBS score ranges from 0 to 56. A higher score indicates an improved ability to balance [22].

The Brunel balance assessment (BBA) has also been used to assess balance ability in patients with stroke. The BBA is divided into three sections—sitting, standing, and stepping—and consists of 12 items. Patients perform three trials for each item and are evaluated for balance ability in turn until the limit of their ability is reached. The BBA score ranges from 0 to 12. The higher the score, the better the ability to balance [23].

The five sit-to-stand (5STS) test, a reliable evaluation used in patients with stroke and associated with dynamic balance and fall risk, was also performed. A standard chair was used for this evaluation, and the patients were instructed not to utilize their arms. The task involved performing five sit-to-stand movements as quickly as possible without using the back for support. Between each repetition, the examiner confirmed that the patient did not use their arms and that they were fully standing and sitting. The 5STS was evaluated using the time recorded by a stopwatch; it started when the patient stood up from the chair and ended when the patient sat down after five repetitions [24].

Gait speed was evaluated using the 10 m walking test (10MWT). It requires a hallway with clearly set distances and a stopwatch. The patients walked at a self-selected speed (comfortable and safe speed), and the examiner walked around them for their safety. To allow for the section of acceleration and deceleration, out of a total of 20 m, the assessment started when the patient’s feet passed the 5 m mark and ended at the 15 m mark. The middle 10 m was identified by a mark on the tape on the side of the corridor. The total time was recorded in m/s [25].

The time up and go (TUG) test, designed to evaluate functional mobility in patients, was conducted. It requires a chair with armrests; a 3 m distance was marked on the floor and a turning point was placed at its end. The test began with the patient seated, followed by standing up and walking a 3 m distance. The patient then returned to the starting point and resumed a seated position. The timing ceased when the patient was fully seated again [26].

The 6-minute walk test (6MWT) was used to assess aerobic capacity and endurance. A 30 m indoor corridor was used, and the starting and return points were marked at both ends of the corridor. The participants were instructed to walk a distance of 30 m from the starting point and return. The examiner walked around while checking the patient’s safety. After 6 min, the participants ceased walking, and the repetitions between the start and return points were tallied to calculate the total walking distance [25,27].

### 2.4. Statistical Analysis

All the statistical analyses were performed using IBM SPSS Statistics for Windows version 25.0 (IBM Corp., Armonk, NY, USA). The Kolmogorov–Smirnov test was employed to determine the normal distribution of the data. Pearson’s correlation coefficient was used to examine the associations among the respiratory, balance, and gait ability variables. Multiple regression models employing a forward selection procedure were applied to identify the most significant respiratory contributors to balance and gait ability. Statistical significance was established at *p* < 0.05.

## 3. Results

The participants’ characteristics are presented in Table 1, and descriptive statistics for the variables are presented in Table 2. All the variables were normally distributed (*p* > 0.05).

Table 3 shows the correlation coefficients between the initial respiratory variables and the balance and gait abilities at discharge. Positive correlations were observed between the TIS and FVC (*r* = 0.625; *p* < 0.001), PEF (*r* = 0.648; *p* < 0.001), and MEP (*r* = 0.502; *p* = 0.004); between the BBS and PEF (*r* = 0.470; *p* = 0.008); between the BBA and PEF (*r* = 0.516; *p* = 0.003) and MEP (*r* = 0.447; *p* = 0.012); between the 10MWT and PEF (*r* = 0.489; *p* = 0.005) and MEP (*r* = 0.566; *p* = 0.001); and between the 6MWT and MEP (*r* = 0.497; *p* = 0.004).

The results of the forward selection regression analyses revealed the following models: Model 1 included the PEF as a predictor variable, explaining 42.0% of the variance in the TIS scores (Table 4; *p* < 0.001). In Model 1, the PEF was a predictor variable accounting for 22.1% of the variance in the BBS scores (Table 4; *p* = 0.008), the PEF was a predictor variable explaining 26.6% of the variance in the BBA results (Table 4; *p* = 0.003), the MEP was a predictor variable accounting for 32.0% of the variance in the 10MWT results (Table 4; *p* = 0.001), and the MEP was a predictor variable explaining 24.7% of the variance in the 6MWT results (Table 4; *p* = 0.004).

The unstandardized and standardized coefficients are listed in Table 5. Using the constant values of the unstandardized coefficients, regression equations were established for each independent variable. The TIS, BBS, BBA, 10MWT, and 6MWT results could be computed using their respective regression equations.

The standardized coefficients (β values) of Model 1 indicated the following influences on the respective variables: the PEF influenced the TIS scores (β = 0.648), the PEF influenced the BBS scores (β = 0.470), the PEF influenced the BBA results (β = 0.516), the MEP influenced the 10MWT results (β = 0.566), and the MEP influenced the 6MWT results (β = 0.497).

## 4. Discussion

In this study, we investigated the respiratory variables (FVC, PEF, MIP, and MEP) associated with balance (TIS, BBS, BBA, and 5STS) and gait (10MWT, TUG, and 6MWT) abilities in patients with stroke. We found that the PEF and MEP play crucial roles at the initial stage for predicting balance and gait ability at discharge from rehabilitation in patients with stroke. 

These findings offer valuable insights into the prediction of balance and gait ability among patients with stroke. Jeong et al. [18] evaluated the relationship between postural control and respiratory muscle strength and identified respiratory function parameters predicting functional outcomes at discharge in 52 patients with subacute stroke. Their study highlighted initial peak cough flow (PCF) and FVC as predictive factors for the final TIS and BBS scores. The congruence between the PCF and PEF in predicting the outcomes in their study aligns with our findings. However, unlike our study, their study emphasized the FVC as a predictor of balance ability at discharge. Jandt et al. [2] evaluated the correlation among trunk control, respiratory muscle strength, and pulmonary function in 23 patients with stroke. They found statistically significant correlations between the TIS and PEF and between the TIS and MEP. Correspondingly, they reported positive correlations between the PEF and MEP with balance ability. A relationship exists among the respiratory variables, balance, and gait, particularly involving the strength of the muscles responsible for forced expiration. However, aside from the PEF, pulmonary function does not seem to be strongly associated with balance or gait ability, which is likely due to the predominant force exerted by the expiratory muscles.

Following a stroke, both the respiratory muscle strength and mobility often decline owing to impaired trunk function [28]. Patients with stroke commonly show characteristics resembling those of restrictive lung disease in contrast to the characteristics of their healthy counterparts [27,29]. This type of pulmonary impairment is characterized by a reduced total lung capacity, which is apparent when the FVC falls below 80% of the predicted normal value during pulmonary function testing. In our study, patients with stroke similarly showed diminished %FVC values, averaging approximately 83%. Teixeira-Salmela et al. [14] showed significant decreases in the MIP and MEP values in patients with chronic stroke compared with the values for healthy age-matched individuals. Similarly, in our study, we found that the respiratory muscle strength values, specifically the MIP and MEP, were below the normal range in patients with stroke. This decline in respiratory muscle strength could lead to respiratory challenges in patients with stroke, thereby impacting cardiopulmonary function and exercise capacity and potentially delaying their participation in rehabilitation programs, ultimately hampering activity and gait performance.

The PEF demonstrated significant correlations with the TIS, BBS, and BBA scores, accounting for 42.0%, 22.1%, and 26.6% of the variance in Model 1, respectively. Gandevia et al. [16] have reported a linkage between respiratory muscle contractions and trunk control. These muscles serve the dual purpose of facilitating breathing and sustaining postural stability during tasks demanding repetitive changes in trunk position. Jandt et al. [2] investigated the relationships among trunk control, respiratory muscle strength, and pulmonary function in patients with stroke. They found significant correlations between the TIS score and the PEF (*r* = 0.489) and between the TIS score and the MEP (*r* = 0.517). Our study validates a relationship between trunk control and expiratory parameters, especially in the PEF. Trunk stability is achieved through proper activation and coordination of the abdominal and vertebral muscles along with the diaphragm and intercostal muscles, which are essential for breathing [30]. This supports the notion that expiratory factors could influence the TIS score by leveraging the strength of the abdominal, internal intercostal, and other axial muscles [2]. Sitting balance and trunk function in stroke patients are related to functional abilities, such as balance [31,32,33]. Hence, it is conceivable that expiratory parameters, such as the PEF, exert an influence on balance assessments in these individuals. Notably, the PEF requires strong expiratory function, engaging key muscles in the expiratory process, such as the abdominal wall muscles (rectus abdominis, transversus abdominis, external oblique, and internal oblique muscles) and the rib muscles (triangularis sterni and internal intercostal muscle) [34]. Consequently, the TIS score, a measure of trunk ability, showed a more significant correlation with other balance assessment results. The lower correlation observed with other balance assessments compared with the TIS score could be attributed to the broader spectrum of balance levels covered by the BBS and BBA, encompassing various aspects of sitting, standing, and stepping. Conversely, all the respiratory variables were insignificant in the 5STS balance assessment, which is likely due to the need for substantial lower-extremity muscle strength (particularly knee extensors) during the transition from sitting to standing, executed without arm and back support [24]. Consequently, employing a multiple regression equation (y = 9.402 + 1.377x) could enable TIS score prediction through PEF assessment. Similarly, PEF assessment could forecast the BBS score using the equation (y = 11.632 + 3.928x), whereas PEF assessment could serve as a predictor for the BBA result via the equation (y = 3.843 + 0.786x).

The MEP was significantly correlated with the 10MWT and 6MWT results of gait ability, accounting for 32.0% and 24.7% of the variance in Model 1, respectively. Trunk dysfunction in patients with stroke can lead to respiratory muscle weakness and limited thoracic mobility. Trunk and neck dysfunction influence gait in patients with stroke [28]. Gait asymmetry and decreased gait speed can become remarkable in patients who have experienced a stroke [35]. Terui et al. [28] revealed the relationship between respiratory function and gait asymmetry in these individuals. They used the symmetry index (SI) to evaluate the temporal asymmetry of gait and the Lissajous index (LI) based on trunk acceleration values to evaluate gait symmetry [36,37]. They showed that the SI was significantly correlated with the expiratory and inspiratory muscle strength (*r* = −0.392, *r* = −0.386, *p* < 0.05, respectively). In addition, the LI was significantly correlated with the percentage of predicted VC (*r* = −0.446; *p* < 0.05). Terui et al. [38] investigated the relationship between gait asymmetry and respiratory muscle strength in patients with stroke. In this study, the LI was significantly correlated with the %PEmax (*r* = −0.478, *p* < 0.05), whereas the LI variation was significantly correlated with the variation in the %PEmax (*r* = −0.546, *p* < 0.05). These data suggest that increasing expiratory muscle strength and improving gait asymmetry are closely related in patients with stroke. The inspiratory muscles include the muscles in the neck and thorax. Conversely, the expiratory muscles contain the muscles of the lower trunk. Therefore, the muscle strength from the neck to the trunk may be related to gait ability and asymmetry. Additionally, the LI was associated with standing balance, and the SI was correlated with gait speed [36,37]. Therefore, it is likely that expiratory muscle strength can influence gait ability in patients with stroke. When assessing gait ability, the MEP showed a significant correlation with the 10MWT and 6MWT results, but not with those of the TUG. In this sense, the TUG test showed a higher correlation with the BBS score than with the gait speed in patients with stroke [26]. The TUG is used to evaluate functional mobility, and it requires complex abilities, such as sitting to standing, turning around, and walking. Consequently, an MEP assessment could predict the 10MWT and 6MWT results through two distinct multiple regression equations ([y = −0.142 + 0.007x] and [y = −20.044 + 2.147x], respectively).

According to the results of this study, initial PEF and MEP values can serve as predictive indicators for balance and gait ability at discharge among patients with stroke. Consequently, it becomes pertinent to assess and track these initial respiratory variables in patients with stroke. 

### Limitations of the Study

This study had some limitations. First, the sample size was deemed insufficient to attain robust statistical power. Circumstances related to coronavirus disease, which led to the early isolation and discharge of several patients along with a dropout rate due to deteriorating conditions, influenced the overall size of the study cohort. Second, in cases of stroke patients with facial weakness unable to maintain a robust oral seal, there is potential for air leakage during both pulmonary function and respiratory muscle strength assessments. Third, the characteristics of patients with stroke, if considered, are insufficient to confirm whether respiratory parameters are independent predictors by adjusting for various confounding variables (such as gender, body mass index, National Institutes of Health Stroke Scale, and modified Rankin Scale). Future studies should aim for larger sample sizes to effectively establish the link between respiratory variables and balance and gait ability in patients with stroke.

## 5. Conclusions

We investigated the influence of respiratory muscle strength and pulmonary function on balance and gait during discharge from rehabilitation for patients with stroke. Notably, the initial respiratory function variables, PEF and MEP, demonstrate the capacity to anticipate balance and gait ability among patients with stroke at the point of discharge. These results underscore the significance of evaluating respiratory muscle strength and pulmonary function during the early stages after ischemic and hemorrhagic strokes as potential prognostic factors for balance and gait proficiency among the patients.

## Figures and Tables

**Table 1:** General characteristics of study participants.

Characteristics	Stroke (n = 31)
Age (years)	68.68 ± 5.85
Height (cm)	163.68 ± 7.89
Weight (kg)	63.42 ± 13.19
Gender (male/female)	25/6 (81/19)
Lesion type (infarction/hemorrhage)	16/15 (52/48)
Time since stroke onset (days)	43.10 ± 17.39
Paretic side (left/right/bilateral)	19/8/4 (61/26/13)
K-MMSE	22.23 ± 4.60

Values are presented as mean ± standard deviation or number (%). K-MMSE: Korean version of the mini-mental state examination.

**Table 2:** Descriptive statistics for variables.

Variables	Mean ± SD
FVC (L)	2.84 ± 0.77
FVC (% predicted)	82.52 ± 18.61
PEF (L/s)	5.87 ± 2.14
PEF (% predicted)	86.32 ± 28.90
MIP (cmH_2_O)	66.81 ± 22.42
MIP (%)	79.81 ± 26.96
MEP (cmH_2_O)	95.84 ± 36.23
MEP (%)	91.19 ± 38.57
TIS	17.48 ± 4.55
BBS	34.68 ± 17.90
BBA	8.45 ± 3.26
5STS (s)	11.39 ± 9.91
10MWT (m/s)	0.54 ± 0.46
TUG (s)	19.93 ± 20.04
6MWT (m)	185.71 ± 156.62

Values are presented as mean ± standard deviation. BBA: Brunel balance assessment; BBS: Berg balance scale; FVC: forced vital capacity; MEP: maximal expiratory pressure; MIP: maximal inspiratory pressure; PEF: peak expiratory flow; SD: standard deviation; TIS: trunk impairment scale; TUG: time up and go; 5STS: five times sit-to-stand; 6MWT: 6 min walk test; 10MWT: 10 m walking test.

**Table 3:** Results of Pearson’s correlation between the initial respiratory variables and the balance and gait variables at discharge.

	FVC	PEF	MIP	MEP
TIS	0.625 *	0.648 *	0.378	0.502 *
BBS	0.232	0.470 *	0.188	0.426
BBA	0.321	0.516 *	0.173	0.447 *
5STS	0.208	0.206	0.217	0.060
10MWT	0.262	0.489 *	0.228	0.566 *
TUG	0.133	0.079	0.143	−0.043
6MWT	0.245	0.435	0.221	0.497 *

BBA: Brunel balance assessment; BBS: Berg balance scale; FVC: forced vital capacity; MEP: maximal expiratory pressure; MIP: maximal inspiratory pressure; PEF: peak expiratory flow; TIS: trunk impairment scale; TUG: time up and go; 5STS: five times sit-to-stand; 6MWT: 6 min walk test; 10MWT: 10 m walking test. According to the Bonferroni adjustment, statistical significance was considered at * *p* < 0.0125.

**Table 4:** Results of forward selection multiple regression analysis for models between initial respiratory variables and the balance and gait variables at discharge.

Dependent Variable	Model	Independent Variable	R^2^	Adjusted R^2^	F	*p*	Durbin–Watson
TIS	1	PEF	0.420	0.401	21.043	<0.001	2.159
BBS	1	PEF	0.221	0.194	8.239	0.008	1.770
BBA	1	PEF	0.266	0.241	10.510	0.003	1.753
10MWT	1	MEP	0.320	0.297	13.674	0.001	2.093
6MWT	1	MEP	0.247	0.221	9.495	0.004	2.014

BBA: Brunel balance assessment; BBS: Berg balance scale; MEP: maximal expiratory pressure; PEF: peak expiratory flow; TIS: trunk impairment scale; 6MWT: 6 min walk test; 10MWT: 10 m walking test. The Durbin–Watson statistic is a test statistic to detect autocorrelation in the residuals from a regression analysis.

**Table 5:** Results of forward selection multiple regression analysis for coefficients of independent variables in models between initial respiratory variables and balance and gait variables at discharge.

Dependent Variable	Model	Independent Variable	Unstandardized Coefficients		Standardized Coefficients	*t*	*p*	95% Confidence Interval for B		Collinearity Statistics	
			B	Standard Error	β			Lower Bound	Upper Bound	Tolerance	VIF
TIS	1	PEF	1.377	0.300	0.648	4.587	<0.001	0.763	1.992	1.000	1.000
BBS	1	PEF	3.928	1.368	0.470	2.870	0.008	1.129	6.727	1.000	1.000
BBA	1	PEF	0.786	0.242	0.516	3.242	0.003	0.290	1.281	1.000	1.000
10MWT	1	MEP	0.007	0.002	0.566	3.698	0.001	0.003	0.011	1.000	1.000
6MWT	1	MEP	2.147	0.697	0.497	3.081	0.004	0.722	3.572	1.000	1.000

BBA: Brunel balance assessment; BBS: Berg balance scale; MEP: maximal expiratory pressure; PEF: peak expiratory flow; TIS: trunk impairment scale; VIF: variance inflation factor; 6MWT: 6 min walk test; 10MWT: 10 m walking test. Collinearity, in statistics, is the correlation between the predictor variables.

## Data Availability

The data that support the findings of this study are available upon request from the corresponding author.
